# Rates of subsequent surgeries after meniscus repair with and without concurrent anterior cruciate ligament reconstruction

**DOI:** 10.1371/journal.pone.0294964

**Published:** 2023-11-28

**Authors:** Joseph B. Kahan, Patrick Burroughs, Logan Petit, Christopher A. Schneble, Peter Joo, Jay Moran, Maxwell Modrak, William Mclaughlin, Adam Nasreddine, Jonathan N. Grauer, Michael J. Medvecky

**Affiliations:** 1 Department of Orthopaedics and Rehabilitation, Yale School of Medicine, New Haven, CT, United States of America; 2 Yale School of Medicine, New Haven, CT, United States of America; Villa Erbosa, Gruppo San Donato., ITALY

## Abstract

**Objectives:**

The purpose of this study was to compare the rates of secondary knee surgery for patients undergoing meniscus repair with or without concurrent anterior cruciate ligament reconstruction (ACLr).

**Methods:**

Utilizing a large national database, patients with meniscal repair with or without concurrent arthroscopic ACLr were identified. The two cohorts were then queried for secondary surgical procedures of the knee within the following 2 years. Frequency, age distribution, rates of secondary surgery, and type of secondary procedures performed were compared.

**Results:**

In total, 1,585 patients were identified: meniscus repair with ACLr was performed for 1,006 (63.5%) and isolated meniscal repair was performed for 579 (36.5%). Minimum of two year follow up was present for 487 (30.7% of the overall study population).

Secondary surgery rates were not significantly different between meniscus repair with concurrent ACLr and isolated meniscus repairs with an overall mean follow up of 13 years (1.5–24 years) (10.6% vs. 13.6%, p = 0.126). For the 2 year follow up cohort, secondary surgery rates were not significantly different (19.3% vs. 25.6%, p = 0.1098). There were no differences in survivorship patterns between the two procedures, both in the larger cohort (p = 0.2016), and the cohort with minimum 2-year follow-up (p = 0.0586).

**Conclusion:**

The current study assessed secondary surgery rates in patients undergoing meniscus repair with or without concurrent ACLr in a large patient database. Based on this data, no significant difference in rates of secondary knee surgery was identified.

## Introduction

Meniscal tears are the most commonly treated knee injury in the United States, with an incidence between 60 and 70 per 100,000, and approximately 850,000 meniscal procedures performed annually [1–4]. The majority of such procedures are meniscal debridement, however in select cases meniscal repair can be considered [5–8]. The durability of such repair procedures has been quoted to be between 84–91% at two years not requiring subsequent surgery [9,10]. In prior studies, a subset of meniscus repairs have been found to be incompletely healed but the patients can remain asymptomatic [11,12]. If there is not biologic healing and further intervention is needed, a meniscectomy is most frequently performed. However, revision meniscus repair, meniscus transplantation and knee arthroplasty can be viable alternatives for the failed meniscus repair, depending upon the status of the articular cartilage [13,14].

Meniscal injury occurs in approximately 50% of patients with an acute anterior cruciate ligament (ACL) injury [15,16]. Meniscal repair performed concurrent with anterior cruciate ligament reconstruction (ACLr) has received specific attention. Some studies have found that healing rates of meniscus repairs with concurrent ACLr are higher than isolated meniscus repair [10,17,18]. This has been postulated to be due to the rich biologic environment created during reconstruction. In their prospective matched cohort study of approximately 1250 patients in each cohort, Wasserstein et al. found higher rates of secondary knee surgery in patients undergoing isolated meniscus repair (16.7%), compared to a combined meniscus repair and ACLr (9.7%) at 2 year follow up [10].

However, other studies of clinical failure rates of isolated repairs and those performed concurrently with ACLr have found them to fare similarly over time [9,19,20]. With minimum follow up of five years, Bogunovic et al. found a failure rate of 12% with isolated repairs and a 18% failure rate with combined ACL reconstructions in a total of 75 patients, although this difference was not statistically significant [19].

Overall, the literature is inconsistent about whether meniscal repair performed concurrently with ACLr has an increased durability than those performed without concurrent ACLr. The goal of the current study was to use a large patient database to estimate the failure rate for a meniscal repair, as reflected by subsequent knee surgery, and compare these rates of secondary knee surgery between isolated meniscus repairs and meniscus repairs with concurrent ACL reconstructions.

## Methods

### Patient cohorts

The large insurance claims PearlDiver (Colorado Springs, CO, USA) 2007–2017 Mariner database, which captures data from approximately 55 million patients, was utilized. An exemption from the institutional review board was obtained, as the database contains only de-identified patient data.

Study patients were identified by Current Procedural Terminology (CPT) codes for meniscal repair (29882 and 29883) with or without concurrent arthroscopic ACLr (CPT code 29888). Both the isolated meniscus repair and the combined meniscus repair and ACLr cohorts were then queried for secondary surgical procedures of the knee within the following 2 years. Secondary surgeries of the knee included those specifically focused on the meniscus injury (repair, meniscectomy, meniscus transplant) and those that could be utilized to treat the symptomatic patient with meniscus deficiency (high tibial osteotomy, unicompartmental knee arthroplasty, and total knee arthroplasty) [**Table 1**].

**Table 1:** Description of study population.

**Meniscus Repair with ACL Reconstruction Procedure Codes**	
**(CPT-29882 or CPT-29883) AND CPT-29888**	
CPT-29882	Arthroscopy knee surgical; with meniscus repair (medial OR lateral)
CPT-29883	Arthroscopy knee surgical; with meniscus repair (medial AND lateral)
CPT-29888	Arthroscopically aided anterior cruciate ligament repair/augmentation or reconstruction
**Meniscus Repair without ACL Reconstruction Procedure Codes**	
**(CPT-29882 or CPT-29883) NOT CPT-29888**	
CPT-29882	Arthroscopy knee surgical; with meniscus repair (medial OR lateral)
CPT-29883	Arthroscopy knee surgical; with meniscus repair (medial AND lateral)
**Description of Secondary Knee Surgery Procedure Codes**	
**Any of the following**	
CPT-27440	Arthroplasty knee tibial plateau;
CPT-27441	Arthroplasty knee tibial plateau; with debridement and partial synovectomy
CPT-27442	Arthroplasty femoral condyles or tibial plateau(s) knee;
CPT-27443	Arthroplasty femoral condyles or tibial plateau(s) knee; with debridement and partial synovectomy
CPT-27445	Arthroplasty knee hinge prosthesis (eg. Walldius type)
CPT-27446	Arthroplasty knee condyle and plateau; medial OR lateral compartment
CPT-27447	Arthroplasty knee condyle and plateau; medial AND lateral compartments with or without patella resurfacing (total knee arthroplasty)
CPT-27457	Osteotomy proximal tibia including fibular excision or osteotomy (includes correction of genu varus (bowleg) or genu valgus (knock-knee)); after epiphyseal closure
CPT-29868	Arthroscopy knee surgical; meniscal transplantation, (medial OR lateral)
CPT-29880	Arthroscopy knee surgical; with meniscectomy (medial AND lateral including any meniscal shaving)
CPT-29881	Arthroscopy knee surgical; with meniscectomy (medial OR lateral including any meniscal shaving)
CPT-29882	Arthroscopy knee surgical; with meniscus repair (medial OR lateral)
CPT-29883	Arthroscopy knee surgical; with meniscus repair (medial AND lateral)

### Data analysis

Data was analyzed in two ways: all patients identified and those who were confirmed to have remained active in the database for a minimum of two years after the index procedure. Confirmed activity was indicated by the patient being seen for any medical condition at or after two years and therefore guarantees two-year survivorship with or without a secondary procedure.

Frequency, age distribution, rates of secondary surgery, and type of secondary procedures performed were assessed and compared using Fisher exact tests and t-tests, as appropriate. Finally, Kaplan Meier Survival curves were created for the larger cohort and the cohort with 2 years of minimum follow up to evaluate isolated meniscus repair and meniscus repair with combined ACL reconstruction. Comparison of the two groups for each of the sets of cohorts was performed with Log-Rank (Mantel-Cox) test.

Stata v.14 (Stata Corporation, College Station, Texas) was used for analysis. Significance was defined with a two-sided alpha level of ≤ 0.05.

## Results

### Study cohorts

**Fig 1:**
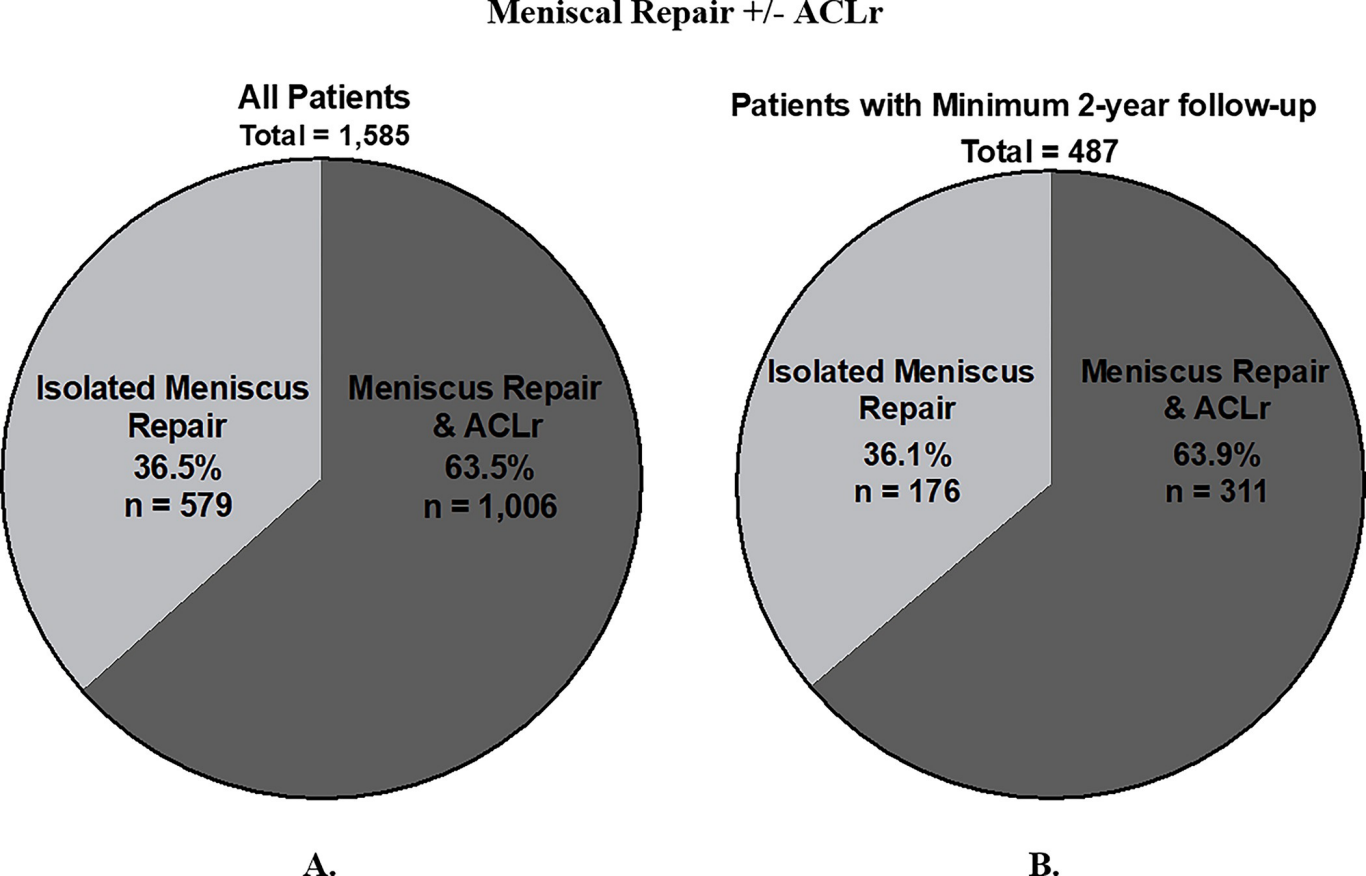
Comparison of study population, patients who underwent isolated meniscus repair versus meniscus repair with ACL reconstruction. Comparison of patients who underwent isolated meniscus repair versus meniscus repair with ACL reconstruction is shown for all patients that met inclusion criteria based on procedural codes and age (A), and that same population after filtering for patients with a minimum of 2-years follow-up (B). There were no significant differences in the frequency of procedure performed between the larger cohort and sub-cohort (p = 0.8756).

In total, 1,585 patients undergoing meniscal repair were identified (**Fig 1**). Isolated meniscal repair was performed for 579 (36.5%) and meniscal repair with ACLr was performed for 1,006 (63.5%). Average follow up was 13 years in the overall cohort, with a range of 1.5 to 24 years. Minimum of two year follow up was present for 487 (30.7% of the overall study population). There was no significant difference in the frequency of procedure performed between the larger cohort and sub-cohort (p = 0.8756).

**Fig 2:**
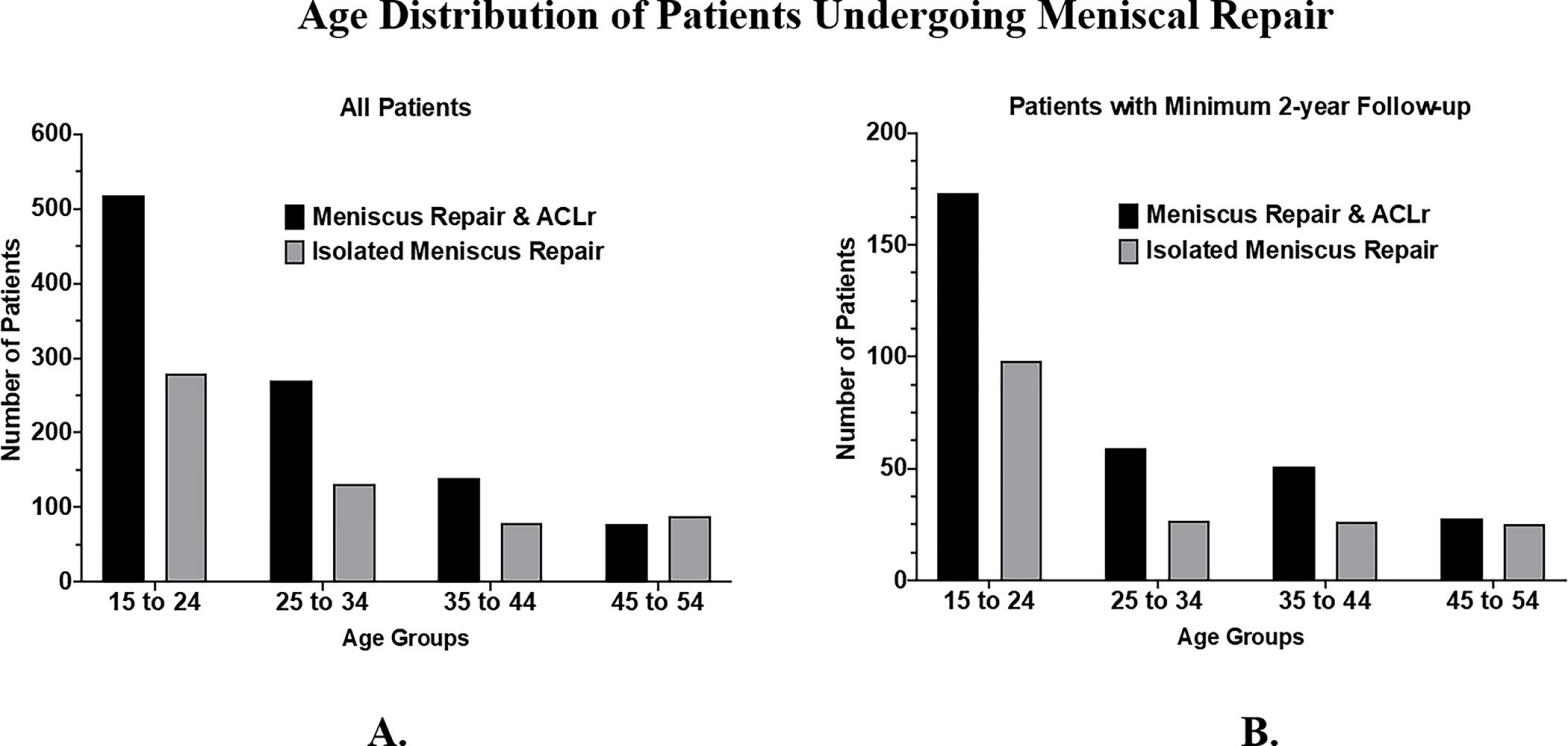
Age distribution of patients undergoing meniscus repair. Comparison of age distributions who patients who underwent isolated meniscus repair to those who underwent meniscus repair with ACL reconstruction (all patients [A], and those with minimum 2 year follow up [B]).

Age distribution of the combined meniscus repair and ACL reconstruction and isolated meniscus repair are shown in **Fig 2** for both the larger cohort (**Fig 2A**), and the cohort with 2 year follow up (**Fig 2B**). The majority of patients were between 15 and 24 years old (50.4%, n = 799). In the larger cohort, a significantly higher proportion of patients in the older age group (45–54) underwent isolated meniscus repair (15.20% vs. 7.75%, p < 0.0001); otherwise, there were no other differences in age distribution. This trend is also present in the smaller study cohort with 2 year follow up (14.20% vs. 9.00%), although the difference was not statistically significant (p = 0.0951).

### Secondary surgeries

**Fig 3:**
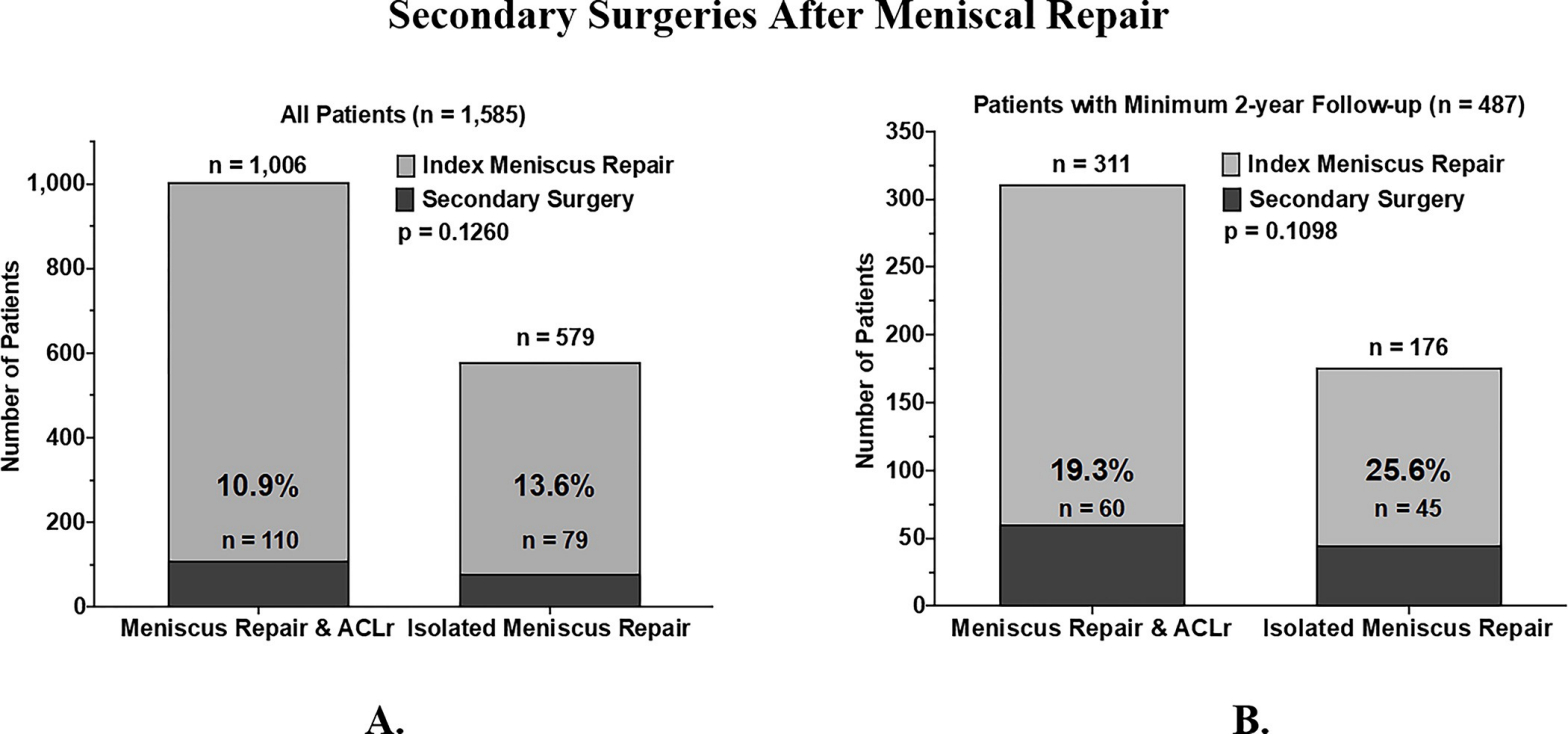
Secondary surgeries after meniscal repair. Secondary surgery rates in patients who underwent isolated meniscus repair versus meniscus repair with ACL reconstruction. Results are shown for all patients (A), and those with a minimum of 2-years follow-up (B). Secondary surgery rate was not significantly different between those undergoing meniscal repair with or without ACL reconstruction (all patients p = 0.1260, those with minimum two year follow up p = 0.1098).

Rates of secondary surgeries were then assessed (**Fig 3**). For the larger cohort, secondary surgeries were performed for 110/1,006 (10.9%) of those with meniscal repairs with concurrent ACLr, as compared to 79/579 (13.6%) of those with isolated meniscal repair (p = 0.126). For the 2 year follow up cohort, secondary surgery rates were also not significantly different (19.3% vs. 25.6%, p = 0.1098).

Of those revision surgeries, approximately 80% were meniscectomies across all patients and cohorts (**Table 2**). There were no differences in type of revision surgery that could be detected between any of the analyzed cohorts. No meniscus transplants or high tibial osteotomies were subsequently needed and between 1–9 patients had a total knee arthroplasty. Because of privacy compliance in the PearlDiver database, any category of patients with less than 10 are not fully available, which is why a more precise reporting of those secondary surgeries is not available for further analysis, nor for sub-analyses based on patient demographics.

**Table 2:** Breakdown of secondary procedures.

**Breakdown of Secondary Procedures after Meniscus Repair & ACLr**			
Code	Description	All Patients (1,006): n (%)	Patients >2 Years Follow-up (311): n (%)
** **	**Total**	**110 (10.9)**	**60 (19.3)**
CPT-29881	Arthroscopy knee surgical; with meniscectomy (medial OR lateral including any meniscal shaving)	75 (68.2)	48 (80.0)
CPT-29882	Arthroscopy knee surgical; with meniscus repair (medial OR lateral)	27 (24.6)	<10 (6.7)
CPT-29880	Arthroscopy knee surgical; with meniscectomy (medial AND lateral including any meniscal shaving)	11 (10.0)	<10 (6.7)
CPT-29883	Arthroscopy knee surgical; with meniscus repair (medial AND lateral)	<10 (1.0)	<10 (6.7)
CPT-27447	Arthroplasty knee condyle and plateau; medial AND lateral compartments with or without patella resurfacing (total knee arthroplasty)	0 (0)	0 (0)
**Breakdown of Secondary Procedures after Isolated Meniscus Repair**			
Code	Description	All Patients (579): n (%)	Patients >2 Years Follow-up (176): n (%)
** **	**Total**	**79 (13.6)**	**45 (25.6)**
CPT-29881	Arthroscopy knee surgical; with meniscectomy (medial OR lateral including any meniscal shaving)	65 (82.3)	39 (86.7)
CPT-29882	Arthroscopy knee surgical; with meniscus repair (medial OR lateral)	12 (15.2)	<10 (4.4)
CPT-27447	Arthroplasty knee condyle and plateau; medial AND lateral compartments with or without patella resurfacing (total knee arthroplasty)	<10 (1.25)	<10 (4.4)
CPT-29880	Arthroscopy knee surgical; with meniscectomy (medial AND lateral including any meniscal shaving)	<10 (1.25)	<10 (4.4)
CPT-29883	Arthroscopy knee surgical; with meniscus repair (medial AND lateral)	0 (0)	0 (0)

Note: Some patients underwent more than one secondary procedure.

**Fig 4:**
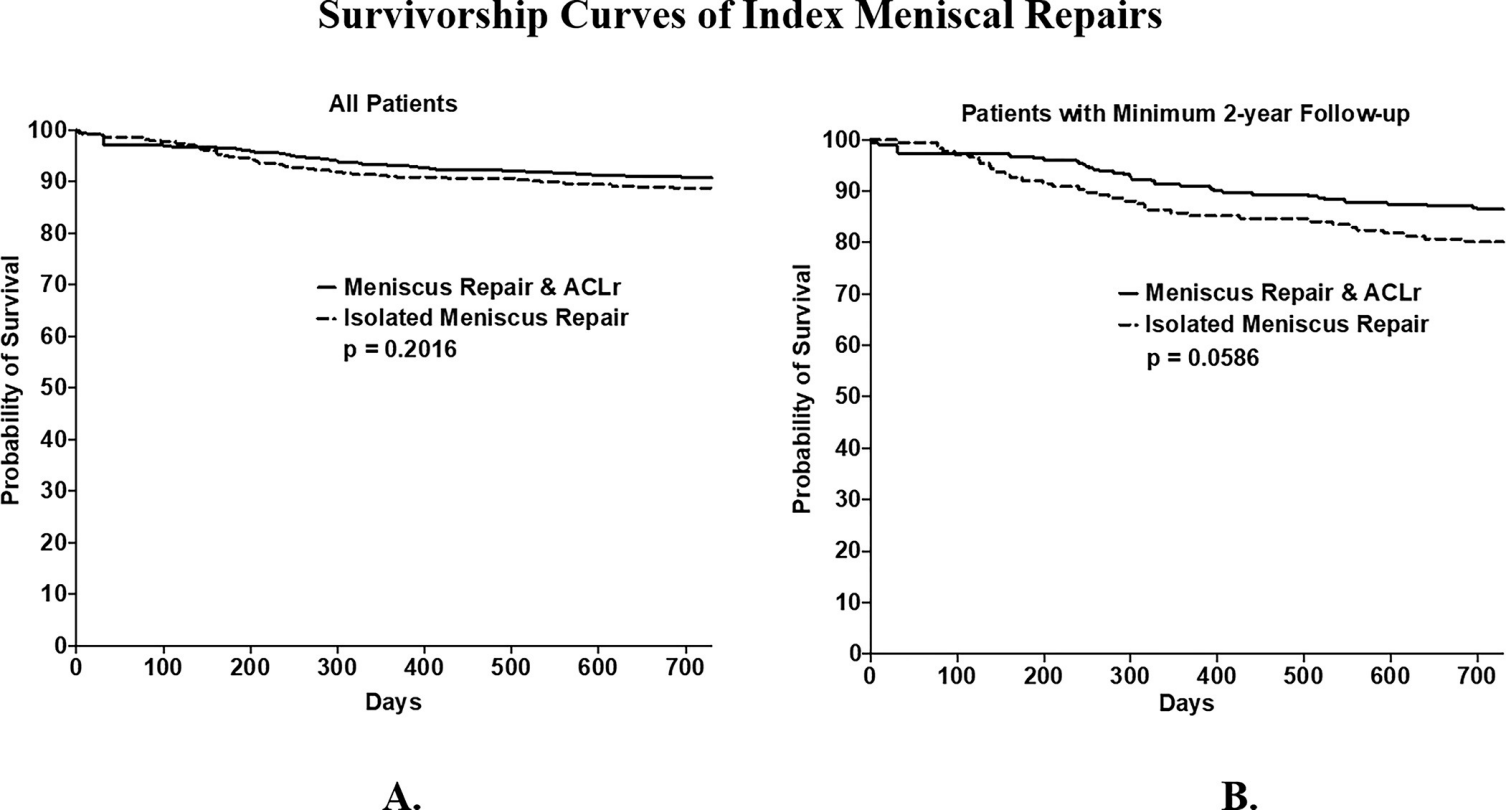
Survivorship curves of index meniscal repairs. Kaplan Meier Survival curve for those undergoing meniscal repair with or without ACL reconstruction for all patients (A) and those with a minimum of two years of follow up (B). Rate of secondary surgery at two years is not significantly different between group (all patients p = 0.2016, those with minimum two years of follow up p = 0.0586).

Kaplan Meier Survivor curves are shown in **Fig 4**. There were no differences in the pattern of survivorship patterns between the two procedures in either cohort set (p = 0.2016 and p = 0.0586).

## Discussion

The importance of maintaining the meniscus when possible is well documented [5–8,10,20,21] Meniscal repair, as opposed to debridement, is thus frequently considered [22–25]. Not only does this scenario become frequently considered in the presence of ACL injury, some [10,18] but not all [9,19–21] studies have suggested that the meniscal repair healing may be improved in the biologic milieu of concurrent ACLr. In the current large database study, we found no significant difference in rates of secondary knee surgery between meniscus repairs performed in isolation and those repaired with combined ACL reconstruction.

In the evaluated dataset, approximately two-thirds of meniscal repairs were performed concurrent with ACLr. Data was analyzed for all patients in the database regardless of follow up, in addition to patients with a minimum of two year follow up, and no differences were found between the larger and smaller cohorts.

There was a predilection of meniscal repairs in conjunction with ACLr in the younger patients. Historically, several studies have reported less favorable results with meniscal repairs in the older population [26,27]. Additionally, older adults with ACL ruptures are more often treated conservatively [28], without surgical intervention, and so the current study’s findings of meniscal repair with concurrent ACLr in younger patients is not surprising.

The rate of secondary surgeries after meniscal repairs were not found to be significantly different between those with or without concurrent ACLr. The average rate of secondary surgeries for the entire cohort was 11.9% and for those with a minimum of two year follow up was 21.6%. These numbers are in line with existing literature [9,10,18–21].

The current study is in line with several studies that have not found differences in secondary surgery rates between meniscal repairs with or without concurrent ACLr [9,19–21]. Bogunovic et al. investigated outcomes of 75 meniscal repairs using an all-inside technique in both isolated meniscus repair and combined meniscus repairs and ACL reconstructions [19]. They found that 84% of patients remained asymptomatic and that there were no significant differences in failures between meniscus repairs with or without concurrent ACL reconstruction [19].

In contrast, the current study is in distinction to other studies that have found differences in secondary surgery rates between meniscal repairs with or without concurrent ACLr [10,18]. Wasserstein et al., in a matched cohort study of approximately 2500 total patients, found higher rates of secondary knee surgery in patients undergoing isolated meniscus repair, compared to combined meniscus repair and ACL reconstruction at 2 year follow up (16.7% vs. 9.7%, p < 0.0001) [10]. Similarly, Ronnblad et al. noted less failure of meniscal repair when simultaneous ACL reconstruction was performed (7% absolute and 42% relative risk reduction of reoperation after 2 years compared with isolated meniscal repair) [29]. However, the present study did note a nonsignificant trend towards less subsequent surgery rates in patients with concurrent ACLr, similar to the studies reviewed.

Despite a lack of published evidence, some authors postulate that the higher healing rates of meniscus repairs with combined ACL reconstruction are due to the rich biologic environment created during reconstruction [10,17,18]. This theory has led to additional techniques, such as notch microfracture with isolated meniscal repair as a means to augment healing via bone marrow stimulation [30]. Fibrin clot, created by spinning autologous blood in a tube until a clot is formed, has also been proposed as an adjunct to healing. Henning et al showed that incorporation of a fibrin clot into an isolated meniscal repair resulted in a failure rate of 8%, compared with 41% without the clot [31]. Other adjuncts such as platelet-rich plasma (PRP) have shown no improvement in self-reported knee function or objective functional testing in patients with combined ACLr and meniscus repair [32]. However, Everhart et al. found that PRP had a protective effect against isolated meniscal repair failure but similarly found no significant benefit when combined with ACLr [33].

When assessing all meniscus repairs in this large database study, we found a 11.9% rate of secondary surgery. For those patients who were followed for a minimum of 2 years, we demonstrated an overall 21.6% rate of secondary knee surgery. Overall, this 21.6% rate of secondary knee surgery at 2 year follow up is consistent with prior literature. Everhart et al., in a study of 235 meniscus repairs treated by a single surgeon, demonstrated a 20.2% failure rate at 5 year follow up [21]. Of those 235 patients, 73% underwent combined meniscus repair and ACL reconstruction, which is slightly higher than the frequency of combined procedures in the current study. Additionally, Everhart et al. demonstrated no difference in failure rates between combined meniscus repairs and ACL reconstructions, and isolated meniscus repairs [21]. In a meta-analysis of meniscus repairs with minimum of 5-year follow, Nepple et al demonstrated a 22.3% to 24.3% failure rate of 566 patients sampled [20]. However, they failed to find a statistically significant difference in failure between combined meniscus repair and combined ACL reconstruction (26.9%) compared to isolated meniscus repairs (22.7%) [20].

Analysis of Kaplan Meier Survival curves demonstrated that there was no significant difference in pattern of failure between combined meniscus repair and combined ACL reconstruction and isolated meniscus repairs. Both cohorts demonstrated a gradual failure over time and not a significant decline at any specific time period.

The limitations of this study are inherent to a database study. The database is only able to capture insured patients, though with both public and private payers and over 55 million lives covered nationally, the large size augments external validity. Additionally, patients that changed insurance status or did not follow up may be lost within the 2 year follow up time period of this study, thus the secondary analysis was performed on those with a minimum 2 year follow up. Furthermore, the inclusion of patients in this study was reliant on the accuracy of the CPT coding, and surgical or clinical data were unavailable, as with most large database studies. This study is unable to stratify the meniscus tear pattern that was repaired, the type of fixation device used to perform the repair as they are indistinguishable based on the CPT code. As the query of CPT codes does not allow for modifiers, laterality data was not available. Further, the evaluation of subsequent surgery rates may be underestimated, as this study purposely did not include subsequent surgeries for microfracture, osteochondral grafting, chondroplasty, or revision ACL repair, as these CPT codes created too much noise when evaluated, and decision was made to only include surgeries directly related to the meniscus and definitive end-points such as total knee replacements. Thus the subsequent surgery rates in this study may be underestimated.

In conclusion, using a large database this study found that meniscus repairs fail at a rate of 21.6% at 2 year follow up, based on the occurrence of secondary knee surgery. Additionally, based on this data, there is no difference between failure rates of meniscus repairs performed alone or with a concurrent ACLr.

## References

[1] Kim S, Bosque J, Meehan JP, Jamali A, Marder R. Increase in outpatient knee arthroscopy in the United States: a comparison of National Surveys of Ambulatory Surgery, 1996 and 2006. J Bone Joint Surg Am. 2011;93(11):994–1000. doi: 10.2106/JBJS.I.01618 21531866

[2] Garrett WE Jr, Swiontkowski MF, Weinstein JN, et al. American Board of Orthopaedic Surgery Practice of the Orthopaedic Surgeon: Part-II, certification examination case mix. J Bone Joint Surg Am. 2006;88(3):660–7. doi: 10.2106/JBJS.E.01208 16510834

[3] Baker BE, Peckham AC, Pupparo F, Sanborn JC. Review of meniscal injury and associated sports. Am J Sports Med. 1985;13(1):1–4. doi: 10.1177/036354658501300101 3838420

[4] Toman CV, Dunn WR, Spindler KP, et al. Success of meniscal repair at anterior cruciate ligament reconstruction. Am J Sports Med. 2009;37(6):1111–5. doi: 10.1177/0363546509337010 19465734 PMC3692358

[5] Baratz ME, Fu FH, Mengato R. Meniscal tears: the effect of meniscectomy and of repair on intraarticular contact areas and stress in the human knee. A preliminary report. Am J Sports Med. 1986;14(4):270–5. 3755296 10.1177/036354658601400405

[6] Lee SJ, Aadalen KJ, Malaviya P, et al. Tibiofemoral contact mechanics after serial medial meniscectomies in the human cadaveric knee. Am J Sports Med. 2006;34(8):1334–44. doi: 10.1177/0363546506286786 16636354

[7] Fairbank TJ. Knee joint changes after meniscectomy. J Bone Joint Surg Br. 1948;30B(4):664–70. 18894618

[8] Ihn JC, Kim SJ, Park IH. In vitro study of contact area and pressure distribution in the human knee after partial and total meniscectomy. Int Orthop. 1993;17(4):214–8. doi: 10.1007/BF00194181 8407035

[9] Westermann RW, Duchman KR, Amendola A, Glass N, Wolf BR. All-Inside Versus Inside-Out Meniscal Repair With Concurrent Anterior Cruciate Ligament Reconstruction: A Meta-regression Analysis. Am J Sports Med. 2017;45(3):719–24. doi: 10.1177/0363546516642220 27159291

[10] Wasserstein D, Dwyer T, Gandhi R, Austin PC, Mahomed N, Ogilvie-Harris D. A matched-cohort population study of reoperation after meniscal repair with and without concomitant anterior cruciate ligament reconstruction. Am J Sports Med. 2013;41(2):349–55. doi: 10.1177/0363546512471134 23263297

[11] Horibe S, Shino K, Nakata K, Maeda A, Nakamura N, Matsumoto N. Second-look arthroscopy after meniscal repair. Review of 132 menisci repaired by an arthroscopic inside-out technique. J Bone Joint Surg Br. 1995;77(2):245–9. 7706339

[12] Rubman MH, Noyes FR, Barber-Westin SD. Arthroscopic repair of meniscal tears that extend into the avascular zone. A review of 198 single and complex tears. Am J Sports Med. 1998;26(1):87–95. doi: 10.1177/03635465980260013301 9474408

[13] Krych AJ, Reardon P, Sousa P, Levy BA, Dahm DL, Stuart MJ. Clinical Outcomes After Revision Meniscus Repair. Arthroscopy. 2016;32(9):1831–7. doi: 10.1016/j.arthro.2016.01.070 27132771

[14] Fuchs A, Kloos F, Bode G, Izadpanah K, Sudkamp NP, Feucht MJ. Isolated revision meniscal repair—failure rates, clinical outcome, and patient satisfaction. BMC Musculoskelet Disord. 2018;19(1):446. doi: 10.1186/s12891-018-2368-0 30577789 PMC6303960

[15] Levy AS, Meier SW. Approach to cartilage injury in the anterior cruciate ligament-deficient knee. Orthop Clin North Am. 2003;34(1):149–67. doi: 10.1016/s0030-5898(02)00065-2 12735207

[16] Fetzer GB, Spindler KP, Amendola A, et al. Potential market for new meniscus repair strategies: evaluation of the MOON cohort. J Knee Surg. 2009;22(3):180–6. doi: 10.1055/s-0030-1247746 19634719 PMC3692350

[17] Espejo-Reina A, Serrano-Fernandez JM, Martin-Castilla B, Estades-Rubio FJ, Briggs KK, Espejo-Baena A. Outcomes after repair of chronic bucket-handle tears of medial meniscus. Arthroscopy. 2014;30(4):492–6. doi: 10.1016/j.arthro.2013.12.020 24680309

[18] Lyman S, Hidaka C, Valdez AS, et al. Risk factors for meniscectomy after meniscal repair. Am J Sports Med. 2013;41(12):2772–8. doi: 10.1177/0363546513503444 24036573

[19] Bogunovic L, Kruse LM, Haas AK, Huston LJ, Wright RW. Outcome of All-Inside Second-Generation Meniscal Repair: Minimum Five-Year Follow-up. J Bone Joint Surg Am. 2014;96(15):1303–7. doi: 10.2106/JBJS.M.00266 25100778 PMC4116561

[20] Nepple JJ, Dunn WR, Wright RW. Meniscal repair outcomes at greater than five years: a systematic literature review and meta-analysis. J Bone Joint Surg Am. 2012;94(24):2222–7. doi: 10.2106/JBJS.K.01584 23318612 PMC3528023

[21] Everhart JS, Magnussen RA, Cavendish PA, et al. Subjective Knee Function and Risk of Failure Are Equivalent for Men and Women at 5 Years After Meniscus Repair. Arthroscopy. 2020;36(3):816–22. doi: 10.1016/j.arthro.2019.09.030 31919022

[22] Stein T, Mehling AP, Welsch F, von Eisenhart-Rothe R, Jager A. Long-term outcome after arthroscopic meniscal repair versus arthroscopic partial meniscectomy for traumatic meniscal tears. Am J Sports Med. 2010;38(8):1542–8. doi: 10.1177/0363546510364052 20551284

[23] Magnussen RA, Mansour AA, Carey JL, Spindler KP. Meniscus status at anterior cruciate ligament reconstruction associated with radiographic signs of osteoarthritis at 5- to 10-year follow-up: a systematic review. J Knee Surg. 2009;22(4):347–57. doi: 10.1055/s-0030-1247773 19902731 PMC3785109

[24] Andersson-Molina H, Karlsson H, Rockborn P. Arthroscopic partial and total meniscectomy: A long-term follow-up study with matched controls. Arthroscopy. 2002;18(2):183–9. doi: 10.1053/jars.2002.30435 11830813

[25] Roos EM, Ostenberg A, Roos H, Ekdahl C, Lohmander LS. Long-term outcome of meniscectomy: symptoms, function, and performance tests in patients with or without radiographic osteoarthritis compared to matched controls. Osteoarthritis Cartilage. 2001;9(4):316–24. doi: 10.1053/joca.2000.0391 11399095

[26] Buseck MS, Noyes FR. Arthroscopic evaluation of meniscal repairs after anterior cruciate ligament reconstruction and immediate motion. Am J Sports Med. 1991;19(5):489–94. doi: 10.1177/036354659101900512 1962715

[27] Eggli S, Wegmuller H, Kosina J, Huckell C, Jakob RP. Long-term results of arthroscopic meniscal repair. An analysis of isolated tears. Am J Sports Med. 1995;23(6):715–20. doi: 10.1177/036354659502300614 8600740

[28] Legnani C, Terzaghi C, Borgo E, Ventura A. Management of anterior cruciate ligament rupture in patients aged 40 years and older. J Orthop Traumatol. 2011;12(4):177–84. doi: 10.1007/s10195-011-0167-6 22075673 PMC3225626

[29] Ronnblad E, Barenius B, Engstrom B, Eriksson K. Predictive Factors for Failure of Meniscal Repair: A Retrospective Dual-Center Analysis of 918 Consecutive Cases. Orthop J Sports Med. 2020;8(3):2325967120905529. doi: 10.1177/2325967120905529 32284936 PMC7137129

[30] Freedman KB, Nho SJ, Cole BJ. Marrow stimulating technique to augment meniscus repair. Arthroscopy. 2003;19(7):794–8. doi: 10.1016/s0749-8063(03)00695-9 12966391

[31] Henning CE, Lynch MA, Yearout KM, Vequist SW, Stallbaumer RJ, Decker KA. Arthroscopic meniscal repair using an exogenous fibrin clot. Clin Orthop Relat Res. 1990;(252):64–72. 2406075

[32] Weldon M, Lowe WR, Lauck K, et al. Does Intra-operative PRP Improve Patient Function & Complication Rates Following ACL Reconstruction With Meniscus Repair? Orthopaedic Journal of Sports Medicine. 2020.

[33] Everhart JS, Cavendish PA, Eikenberry A, Magnussen RA, Kaeding CC, Flanigan DC. Platelet-Rich Plasma Reduces Failure Risk for Isolated Meniscal Repairs but Provides No Benefit for Meniscal Repairs With Anterior Cruciate Ligament Reconstruction. Am J Sports Med. 2019;47(8):1789–96. doi: 10.1177/0363546519852616 31166699

